# Clips closure versus endoloop ligation in laparoscopic appendectomy: a systematic review and meta-analysis of comparative studies

**DOI:** 10.1097/MS9.0000000000001260

**Published:** 2023-09-06

**Authors:** Samuel Ho Ting Poon, Sui Yuen Law, Alex Ting Yeung Lai

**Affiliations:** Department of Surgery, Pamela Youde Nethersole Eastern Hospital, Hospital Authority, Hong Kong

**Keywords:** appendicitis, clips, endoloop

## Abstract

**Introduction:**

Appendiceal stump closure (ASC) is a key step in performing laparoscopic appendicectomy. Currently, there is no gold standard method to achieve this goal. The ideal method should be safe, easily available, and have a short learning curve. Out of all those appendiceal stump closure methods, the use of hem-o-Lok demonstrates its feasibility in replacing the traditionally used endoloop. In this systematic review and meta-analysis, the authors aim to review the currently available evidence addressing the topic of interest.

**Method:**

The PubMed and Embase databases were searched with the paired search terms appendicitis, clip, and endoloop by two authors separately. The quality of the randomized controlled trials was assessed with the Cochrane risk of bias tool, and the quality of the observational studies was assessed with the Newcastle-Ottawa scale. Meta-analysis was conducted with Cochrane Review Manager version 5.4.

**Result:**

Eighteen studies were included for quantitative analysis. The appendiceal stump closure time was shortened by 2 min 7 s using a hem-o-lok with 95% CI 1 min 48 s–2 min 26 s, *p* less than 0.00001. The pooled results of 6 randomized controlled trials demonstrated a statistically significant reduction in operative time of 5.15 min from adopting the hem-o-lok approach (*p*=0.001, 95% CI −2.05 to −8.24 min). Both endoloop and hem-o-lok demonstrated a comparable postoperative hospital stay and infective complication profile.

**Conclusion:**

The application of Hem-o-Lok demonstrates a comparable to endoloop ligation in terms of operative time and a potential benefit on the complication. When considering financial and technical aspects, it serves as an alternative to endoloop.

## Introduction

Highlights

- The use of Hem-o-lok improves the operative time for laparoscopic appendicectomy.
- The application of Hem-o-lok provides a comparable and sound complication profile as in the application of endoloop.
- The use of Hem-o-lok can significantly reduce the operative cost for operation.
- The use of Hem-o-lok can significantly reduce the development of postoperative intra-abdominal collection.

One of the most commonly encountered acute surgical conditions is acute appendicitis. Worldwide incidences range from 7.5 to 22.71 per 10 000, and the lifetime risk is ~16.3%^1,2^. While conservative management with antibiotics is accepted by the American College of Surgeons as a first-line treatment, appendicectomy remains the mainstay of operative treatment in view of the high recurrence rate of the disease with conservative treatment^3,4^. In recent decades, laparoscopic appendectomy has replaced traditional open appendectomy^5–8^. One of the key steps in performing laparoscopic appendicectomy is secure closure of the appendiceal stump since insecure closure could lead to stump leakage, which may result in intra-abdominal collections. The appendiceal stump is traditionally closed with endoloop ligatures, while stapling devices have become favored in recent years. Alternative stump closing devices, including titanium clips and hem-o-lok, have also been discussed. The usage of hem-o-lok in closing appendiceal stumps was first described in 2006 and 2007 by Jenwitheesuk and Hassen *et al.* ^9,10^. With the advantages of being biologically inert and radiolucent, low cost, and having a short learning curve in applications, hem-o-lok is in favour as an alternative to the traditionally used endoloop. The 2020 update of the World Society of Emergency Surgery (WSES) Jerusalem Guidelines recommends the use of endoloops/suture ligation or polymeric clips for stump closure for both adults and children with either uncomplicated or complicated appendicitis^11^. Nevertheless, whether the use of hem-o-lok is superior to loop ligation remains controversial. Various studies, including observational studies and randomized controlled trials, have been performed in the past 15 years to investigate the feasibility of hem-o-lok in replacing endoloop in laparoscopic appendicectomy. This study aims to comprehensively evaluate the evidence on clinical application and analyze the operative outcome with the use of hem-o-lok.

## Method

### Inclusion criteria

Studies that met the following criteria were included: (1) uncomplicated acute appendicitis (excluding perforation and intra-abdominal abscess); (2) full article published in English; and (3) randomized controlled trials (RCTs) and observational comparative studies.

### Exclusion criteria

Studies were excluded if they had the following: (1) studies that focused solely on the paediatric population (subjects’ age < 18); (2) noncomparative studies; (3) open converted cases involved; (4) single port laparoscopic appendicectomy involved; and 5) interval appendicectomy cases.

### Data searches and quality assessments

Embase (1980-) and PubMed databases were searched by two authors, with the search date set as the 5 August 2022. The MeSH terms Appendicitis, Clip, and Endoloop were used for the literature search through the two search engines. The search process was conducted independently, and the findings were entered into a preset Excel document. The authors subsequently combined the search results, and duplications were removed. The literature was independently assessed by the authors and subsequently reviewed together. Additional studies identified from the reference list of included studies were also included. Consensus was achieved on the inclusion of articles. Quality assessments were performed by two authors. The primary outcome was the operative time involved and measured. Length of hospital stay, total time taken for the appendiceal stump closing process, and complications associated with the two treatment modalities were measured as secondary outcomes. This review was performed in line with PRISMA and AMSTAR.

### Statistical analysis

Review Manager (RevMan) [Computer program]. Version 5.4. The Cochrane Collaboration, 2020, was used to evaluate the study results and construct forest plots and funnel plots. The risk ratio (RR) for developing complications in the Hem-o-Lok group compared to the double loop ligation group was employed to evaluate the outcomes of the two interventions. The 95% CI) were used to evaluate the statistical significance of the RR. An RR greater than one dictates a superior outcome for the double loop ligation group and vice versa. The value of RR was considered statistically significant at the *P*=0.05 level. Heterogeneity was accounted for by the I^2^ test. Significant heterogeneity was defined as I^2^ greater than 50%. The random effect model of the Mantel-Haenzel method was used to analyze dichotomous data for an I^2^ value greater than 50%^12^. The analysis of continuous data employed the random effect model of inverse variance due to the great heterogeneity among studies^13^. The fixed effect model was used for analyzing non-heterogeneous data. Publication bias was evaluated by visual evaluation of the funnel plots.

## Result

### Search results

**Figure 1:**
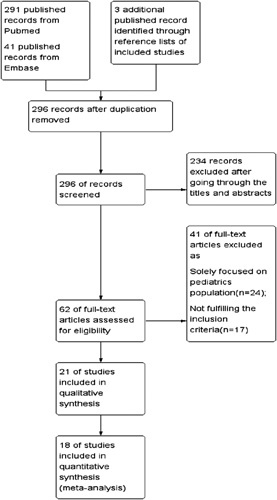
PRISMA flow chart of study selection. PRISMA, Preferred Reporting Items for Systematic Reviews and Meta-Analyses.

The search result was demonstrated in the form of a Prisma flow chart (Fig. 1). Two hundred ninety-one literature records were identified from PubMed, and another 41 articles were found with the Embase search engine. Three additional studies were identified from the reference onlists of the included articles. Thirty-nine duplicated records were removed. Two hundred thirty-four studies out of the 296 studies were excluded after reviewing the titles and abstracts. The remaining 62 papers were included for full article reviews, with 21 studies identified for qualitative synthesis. Eighteen of the 21 studies were included in the quantitative analysis.

### Study characteristics

Among the 18 studies included in the meta-analysis, 6 were randomized controlled trials, including 459 patient records with 230 from the endoloop arm. These studies were conducted between 2012 and 2021. Another 6 prospectively collected cohort studies were included with a sample size of 533. Among the 533 patient records, 275 patients were from the Endoloop arm. The remaining 6 studies were retrospective cohort studies with a sample size of 1593, including 783 patients from the endoloop group. The features of the analyzed literature are shown in Table 1. Quality assessments were performed with the Cochrane risk of bias tool for randomized control trials, while the Newcastle-Ottawa scale was employed to evaluate the quality of the included observational studies. The results are shown in Table 2 and Table 3.

**Table 1:** Illustrates the features of included studies for meta-analysis.

References	Year of publication	Study design	Result	Sample size	Remarks
Delibegović *et al.* ^14^	2009	Prospective cohort	Operative time (min) 47.1±6.7 (EL) 38.7±5.0 (HL) Hospital stay (days) 2.2±0.4 (EL) 2.2±0.4 (HL) Intraoperative Complications 0 (EL) 1 (HL) No postoperative complication	EL 24 HL 28	Cost of 3 hem-o-loks versus 3 endoloops are € 76.9 and € 88.5, respectively
Delibegovic^15^	2012	RCT	Operative time (min) 46.00±7.07(EL) 42.83±6.52 (HL) Hospital stays (days) 2.07±0.45 (EL) 2.03±0.41 Intraoperative Complications 0 (EL); 2 (HL) No postoperative complication	EL 30 HL 30	A single hem-o-lok was applied for the HL arm. 2 patients from the hem-o-lok group developed intraoperative bleeding and managed laparscopically.
Jenwitheesuk *et al.* ^16^	2012	Retrospective cohort	Operative time (min) (Range) 38 (16–90)(HL) 66 (25–130) (EL) Hospital stay (h) (Range) 76 (38–174) (EL) 60 (32–108) (HL) Intra-abdominal collection 0 (EL) 1 (HL) Wound infection 1 (EL) 1 (HL)	EL 23 HL68	Insufficient data for published to include for meta-analysis of hospital stays and operative time analysis
Lucchi *et al.* ^17^	2012	Retrospective cohort	Operative time (min) (Range) 40.5 (10–120) (EL)36.4 (15–110) (HL) Hospital stay (mean) (SD not a/v) 1.20 (EL) 1.23 (HL) Postop Complication 1 SB adhesion in HL group Infective complications 2 (EL) 2 (HL)	EL 158 HL 121	Insufficient data published to be included for meta-analysis of hospital stays and operative time analysis Cost reported for each Endoloop is 92 euros while cost for each hem-o-lok is 8euros
Colak *et al.* ^18^	2013	RCT	Operative time (min) 75.4± 23 (EL) 64.7± 19.2 (HL) Hospital stay (days) 2.5± 2.5 (EL) 2.1±0.7 (HL) Intrabdominal abscess 0 (EL) 1 (HL) Postoperative infective complications 1 (EL) 2 (HL)	EL 27 HL 26	Conducted in Turkey Charge of 3 Endoloop are 120USD and cost for 3 hem-o-lok clips were 30USD
Hue *et al.* ^19^	2013	Prospective cohort	Postop hospital stay 5.2±1.6(EL) 5.3±2.3 days (HL) Complication infective complication 2(EL) 1 (HL) No intraoperative complication	EL 66 HL 39	Operativeve time is not available Conducted in Korea Costs of 3 hem-o-loks and 3 double endoloop were $14.6 USD and $68.2 USD respectively
Sadat-Safavi *et al.* ^20^	2016	RCT	Operative time (min) 23.31±3.5 (EL) 21.53±2.6 (HL) Hospital stay (days) 1.63 for both group Complications Intraoperative complications Clip slippage 0 (EL) 1 (HL) Infective Complications 1 (EL) 0 (HL) None of them developed stump leakage	EL 38 HL 38	Insufficient data published to be included in the meta-analysis of hospital stay
Soll *et al.* ^21^	2016	Retrospective cohort	Operative time 67 (EL) 61.5 (HL) Complication rate Intraoperative complications 2 (EL) 4 (HL) Intra-abdominal abscess 15 (EL) 5 (HL) Infective complications 3 (EL) 2 (HL)	EL 378 HL 435	The largest scale study identified Hem-o-lok ligation resulted in a reduced rate of intra-abdominal surgical abscesses as compared to the application of endoloops Total cost for appendectomy using hem-o-lok clips costed 1993 € and with endoloops costed 2192 €.
Şimşek *et al.* ^22^	2017	Prospective cohort	Operative time (min) 50±10.6 (EL) 40±12.6 (HL) Postop hospital stay (days) 2.23±1.39 (EL) and 1.98±1.42 (HL) No complication developed in both arms	EL 30 HL 30	Provided measurements on the time taken for obliterating the appendiceal stump Ligation using a single HL or EL
Oz *et al.* ^23^	2017	Prospective cohort	Operative time 53.8 ±1.5 (EL) 42.5±1.3 (HL) Postop hospital stay 2±0.2 (EL) 2.1±0.2 (HL) Complication profile No intraoperative complication Postoperative infective complication 2 (EL) 2 (HL)	EL 30 HL 36	Total cost with the use of EL and HL were $1170.8 and $1094, respectively Per cost of HL was $30, while that of EL was $60
Delibegovic *et al.* ^24^	2018	RCT	Operative time (min) 46.0±7.07 (EL) 42.83±6.52 (HL) Hospital stay (days) 2.17±0.70 (EL) 2.23±0.78 (HL) No complication developed in both arm	EL 30 HL 30	It compares four groups of ACS methods, namely Endoloop, Stapler, Hem-o-lok, and Titanium DS clip. One HL costs 7.5 €, PDS endoloop costs 36.99€, and Vicryl Endoloop costs 38.10
Wilson *et al.* ^25^	2018	Retrospective cohort	Operative time (min) (range) 68 (25–160) (EL) 59 (20–175) (HL) Postop hospital stays (days) (range) 2.3 (0–8) (EL) 2.4 (0–13) (HL) Postop complication Infective complication 1 (EL) 1(HL) Port site Hernia 1(EL) 1(HL) Ileus 2(EL) 0(HL) No intraoperative complication	EL 78 HL 47	The use of polymeric clips costs £21 compared with £49 for endoloops per operation Insufficient data published to be included for meta-analysis of hospital stay and operative time
Bhabhor *et al.* ^26^	2019	Prospective cohort	Postop hospital stay 3.4±1.06 (EL) 3.1±0.56 (HL) Complication 3 (EL) 0 (HL)	EL 35 HL 35	No date provided on the mean operative time It suggested that it is more feasible for surgeons to use polymer clips than endoloops to close appendicular stump from surgeon involved in the study
Ihnát *et al.* ^27^	2021	RCT	Operative time (min 45±12.0 (EL) 37.9±12.5(HL) Hospital stays (days) 3.6±1.8 (EL) 3.6±1.7 (HL) No intraoperative complication for both groups Postoperative complications Superficial surgical site infection 4 (EL) 3 (HL) Deep surgical site infection 1 (EL) 1 (HL)	EL 60 HL 60	It compared endoloop, hem-o-lok, and stapler technique and proposed hem-o-lok as the technique of choice for ACS Direct costs for operation are 1705.4±500.9 for endoloops, 1624.9±768 for hem-o-lok and 2120±435 for stapler.
Jan *et al.* ^28^	2021	RCT	Operative time (min) 34.8±8.45 (EL) 25.2±6.08 (HL) Postoperative hospital stay (H) 30 (EL) 26 (HL) Infective complications 3 (EL) 1 (HL)	EL 45 HL 45	The random sequence generation is not well described Insufficient data provided for pooled analysis of postoperative hospital stay.
Erdoğan *et al.* ^29^	2021	Retrospective cohort	Operative time (Min) (Range) 40 (27–63) (EL) 40 (17–85) (HL) Length of hospital stay (days) 1 (range: 1–2) 1 (range: 1–7) Infective complication rate 2 (HL) 3 (EL)	EL 39 HL 37	Insufficient data published to be included for meta-analysis of hospital stay and operative time The endoloop used is hand-made endoloops rather than the standard commercially available endoloop
Vinod *et al.* ^30^	2022	Prospective cohort	Operative time (Min) 50.83±10.5 (EL) 40.3±12.3 (HL) Postop hospital stays (Days) 3.1±0.8 (EL) 2.7±0.9 (HL) No complication developed	EL 90 HL 90	Costs for using hem-o-loks and Endoloops are Rs. 310±Rs. 76 and Rs. 630±Rs. 118, respectively
Mikail *et al.* ^31^	2022	Retrospective cohort	Operative time (min, mean±SD) 54.1±25.3 (EL) 55.5±22.6 (HL) Hospital stay (days, mean±SD) 2.82±2.69 (EL) 2.29 ±1.86) (HL) Intraoperative Complications 0 (EL) 1 (HL) Postoperative complication 17(EL) 7 (HL)	EL 107 HL 102	Costs for using hem-o-loks and endoloops are 58 and 395 Turkish Liras, respectively

EL, Endoloop; HL, Hem-o-lok; Postop, postoperative; RCT, randomized controlled trial.

**Table 2:**
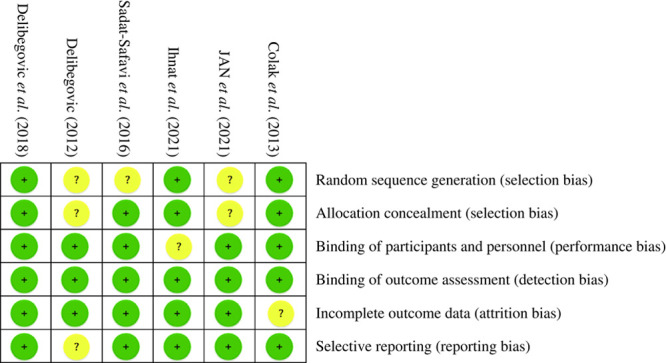
Table showing cochrane risk of bias tool for assessing clinical trials.

**Table 3:** Table showing Newcastle-Ottawa Scale assessment tool for quality of cohort studies.

Newcastle-Ottawa Scale assessment				
References	Selection	Comparability	Outcome	Study quality
Delibegović *et al.* ^14^	****	*	***	Good
Jenwitheesuk *et al.* ^16^	****	*	***	Good
Lucchi *et al*.(2012)^17^	***	*	***	Good
Hue *et al.* ^19^	**	*	***	Fair
Soll *et al.* ^21^	****	**	***	Good
Şimşek *et al.* ^22^	***	*	***	Good
Oz *et al*.[^23^]	***	*	***	Good
Wilson *et al.* ^25^	****	**	***	Good
Bhabhor^26^	****	*	****	Good
Erdoğan *et al.* ^29^	****	**	***	Good
Vinod *et al.* ^30^	****	*	***	Good
Mikail *et al.* ^31^	****	**	***	Good

### Critical appraisal

None of the RCTs were double-blinded studies limited by the nature of the surgery. All the studies provided a clear presentation of the outcomes, as the data were retrieved from surgical records and patient clinical data. The comparators are simple and can be measured easily, which minimizes the chance of missing data and the risk of loss in follow-up. However, the quality of RCTs is limited by an unclear random sequence generation process for some of them, namely, Delibegovic (2012), Sadat-Safavi and colleagues (2016), and Jan and colleagues (2021). There is a substantial difference noted in the operative time between different studies. However, the mean difference between the two comparative arms is comparable, and it is reasonable to attribute the difference to the use of the investigated methods, which makes the comparison in operative time feasible. The quality of the included observational studies is promising, with all of them demonstrating a comparable patient demographic profile and a small chance of missing patient records.

### Analysis results

#### Appendiceal closure time

**Figure 2:**
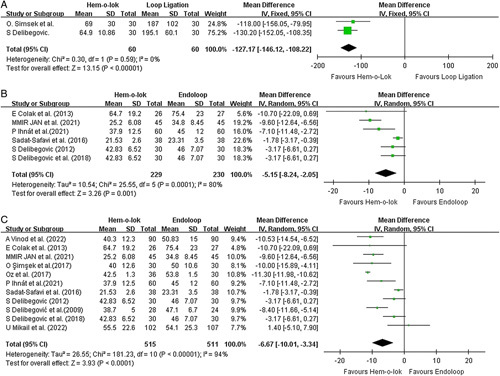
Forest plots illustrate the analysis of operative time. (A) demonstrated the pooled analysis of time taken for appendiceal stump closure (ASC). (B) illustrating the analysis of operative time with randomized controlled trials (RCTs) only. (C) illustrating the analysis of operative time for all the included studies.

The focus of this meta-analysis is to investigate the time involved in closing the appendiceal stump, which, in turn, shortens the operative time. Simsek and colleagues and Delibegovic provided ideas on the time taken to close the appendiceal stump. A total of 120 patient records were included for pooled analysis. These two studies demonstrated a homogenous result that favored Hem-o-lok in shortening the closure time with a mean difference of 2 min 7 s (95% CI 1 min 48 s–2 min 26 s). The result was homogenously significant, with an I^2^ value of 0% and *P* less than 0.00001. (Fig. 2A)

#### Total operative time

Analyses were conducted to evaluate the results of RCTs, and the total results of the studies were included separately. The pooled result of the six included RCTs demonstrated a statistically significant reduction in operative time of 5.15 min from adopting the hem-o-lok approach (*P*=0.001, 95% CI −2.05 to −8.24 min). Significant heterogenicity was noticed with an I2 of 80%; thus, a random effect model was chosen for the analysis (Fig. 2B). A funnel plot was evaluated and showed no significant publication bias. The analysis of the total results included 11 studies with a sample size of 1026 (Fig. 2C.) This demonstrates the superiority of hem-o-lok over endoloop by shortening the operative time by 6.67 min (*P* <0.0001. 95% CI −3.34 to −10.01 min). Heterogenicity was noticed with an I^2^ of 94%.

#### Infective complications

**Figure 3:**
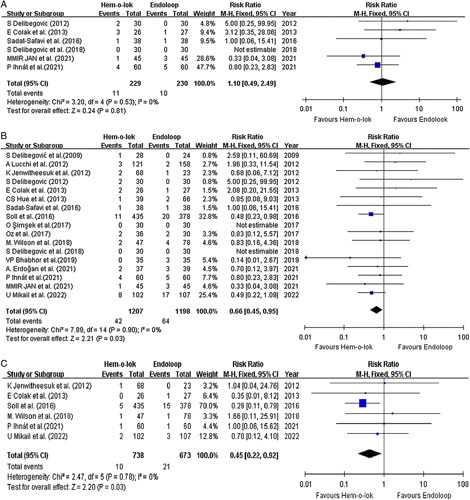
Forest plots demonstrate the analytical result of the complication profile. (A) illustrating the result focusing solely on randomized controlled trials (RCTs). (B) illustrating the result of the overall analysis of all the included studies. (C) illustrating the result of postoperative intra-abdominal collection.

Infective complications included all complications related to infection, including intra-abdominal collections and deep and superficial surgical site infections. The analysis results of the six RCTs demonstrated a comparable and homogenous result for infective complications for the two study arms (Fig. 3A). Eleven patients out of the 229 from the hem-o-lok arm developed infective complications, contrary to 10 out of 230 from the Endoloop arm. The calculated risk ratio was 1.1 with no statistical significance (*P*=0.81 95% CI 0.49–2.49; I^2^=0%). The analysis for both RCTs and observational studies included 17 studies with 1207 patients from the Hem-o-lok group and 1198 patients from the Endoloop group (Fig. 3B). A statistically significant difference was noted in favour of Hem-o-lok (RR=0.66, *P*=0.03 95% CI 0.45–0.95; I^2^ =0%)

#### Postoperative intra-abdominal collection and leakage

Further analysis of 6 studies, including 2 RCTs, found 31 patients who developed postoperative intra-abdominal collection. Ten out of that 31 patients were operated with the hem-o-lok approach (Fig. 3C). Statistical analysis found a superior outcome with the application of hem-o-lok clips. (RR: 0.45 (95% CI 0.22–0.92), *P* = 0.03, I^2^=0%) There were only three studies which documented the result of stump leakage^20,29,31^. None of them was reported to constitute statistical analysis.

#### Hospital stay

**Figure 4:**
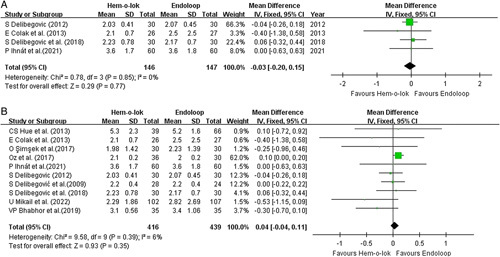
Forest plots illustrate the analysis of postoperative hospital stay between the Hem-o-lok group and Endoloop group. (A) illustrating the analysis of randomized controlled trials (RCTs) only. (B) illustrating the analysis of all included studies.

Postoperative hospital stay was compared between the study arms. Pooled analysis of 4 RCTs including a total of 293 patients from 4 studies demonstrated a homogenous and comparable profile (Fig. 4A). The mean difference in postoperative hospital stay between the two groups was −0.03 days, without any statistical significance (*P*=0.77, 95% CI −0.2 to 0.15 I^2^=0%). The overall meta-analysis of 11 studies provided a sample size of 1035 patients, with 506 from the Hem-o-lok group (Fig. 4B). There was no significant difference in postoperative hospital stay between the two closure methods (mean difference=−0.1 day *P*=0.10, 95% CI −0.24 to 0.05 day, I^2^=51%).

#### Sensitivity test

Sensitivity tests were performed by alternatively removing an individual study from the pooled analysis. In both analyses of RCTs and all the studies, the superior outcome of hem-o-lok in shortening the operative time remains statistically significant with sensitivity tests regardless of which individual study is excluded. The effect of the two-closure method on overall infective complications remains comparable for the analysis of RCTs during the sensitivity test. The result becomes insignificant for the overall analysis when either the study by Soll and colleagues or the study by Mikail and colleagues is removed. The analysis on postoperative intra-abdominal collection becomes insignificant after removing Soll and colleagues It remains significant by alternatively removing other studies. Future large-scale studies may provide further insight into the complication profile of the two appendiceal stump closure (ASC) methods. The difference in postoperative hospital stay remained comparable in the sensitivity test.

## Discussion

Appendicectomy represents one of the most commonly encountered surgical procedures. Since the introduction of the laparoscopic approach (LA), it has gained favour over the conventional open approach in resecting the appendix owing to several advantages, namely, fewer wound infections, shorter length of hospital stays, and early return to normal activity^5,7,8^. Skeletonization of the appendix, controlling the appendiceal artery, and ligating the appendiceal stump are the key steps of laparoscopic appendicectomy. Endoloops and staplers are commonly adopted in closing the base of the appendix. In the early days when LA was first introduced, endoloop ligatures were used to provide a convenient way to obliterate the stump^32,33^. Nevertheless, the applications of endoloops face several limitations, including the learning curve of usage, the chance of slippage of the loop, extra manipulation of the appendix stump, and the chance of cut-through. Since the introduction of laparoscopic linear staplers, their usage in LA has been advocated in view of their safety profile in closing the stump, especially for difficult cases such as perforation at the appendiceal base^34,35^. However, the use of endoscopic staplers is limited by their cost and the involvement of larger working ports. Some have also proposed that metal clips from the stapler may lead to adhesion-related small bowel obstruction. These hinder the applications of linear staplers for ASC, and a gold standard has yet to be established.

The authors believe the best closure method should be safe, economically friendly, easily available, and have a short learning curve. Various alternative options, including metal clips and sutureless energy devices such as Ligasure and Hem-o-lok, have been proposed to close the appendiceal stump^36^. When compared with metal clips, Hem-o-lok provides the advantage of radiolucency, which will not affect future radiological studies on the abdomen. Since the introduction of Hem-o-lok closure by Hassen and colleagues and Jenwitheesuk, several cohort studies and clinical trials have been performed to examine the feasibility of Hem-o-lok in replacing endoloop^9,10^. Hem-o-loks are polymeric clips that are commonly used in closing the cystic duct stump in cholecystectomy and controlling major vessels during laparoscopic procedures. It is easily accessible and available compared with the tailor-made DS appendicectomy metal clip.

When compared with traditional endoloop closure, hem-o-lok also demonstrated several advantages, namely, ease of application, objective measurement in successful closure, and lower cost of equipment. While the failure of hem-o-lok closure will immediately lead to slippage, the tension required for successful ASC with an endoloop is rarely measured intraoperatively. The slippage serves as an objective means to check the successful closure. The hem-o-lok comes in a package of six, with one package costing approximately half of that of one endoloop in our locality. This turns into a 10-fold difference in the total cost of applying three hem-o-loks versus three endoloops. This is consistent with the cost of surgical materials, as reported by Lucchi *et al.* ^17^. Bhabhor^26^ further demonstrated the ease of application for hem-o-lok versus endoloops .

### Stump leakage

Apart from the learning curve and the cost of surgical material, the success rate in achieving appendiceal stump closure is another major concern. A failure will lead to stump leakages and postoperative intra-abdominal collection and associated morbidities. However, the assessment of postoperative stump leakage is technically difficult. Major leakages requiring reoperations can be detected in the subsequent surgeries. On the contrary, minor leakages leading to abscess formations not requiring reoperation are difficult to be differentiated from those formed secondary to ineffective decontamination of the surgical field. Only three studies have included the incidence of leakage, without documenting the method of measurement. Given that the techniques in decontamination of surgical field were the same between two groups of patients, the development of postoperative intra-abdominal collections serves as a reasonable indirect method to monitor stump leakage. In this analysis, we demonstrate a significant reduction in postoperative collection with using Hem-o-lok.

### Safety and applicability

Although previous studies have demonstrated the advantage of applying hem-o-loks, its use is limited by the size of the appendiceal stump. Currently, the largest hem-o-lok on the market is 16 mm in inner diameter. If the stump of the appendix is dilated to a size larger than 16 mm, the application is unlikely to be successful. Furthermore, this study mainly focuses on the application in uncomplicated cases in which its applicability in complicated cases remains uncertain. Future trials may investigate the feasibility of hem-o-lok to serve as a salvage procedure for those with difficulty in applying endoloops and the safety profile for hem-o-lok versus stapler in noncomplicated cases. While both endoloop and hem-o-lok usage demonstrates safe intraoperative complication profiles with no cutting-through incidence documented, concern regarding adhesion between surgical material used to close the stump and surrounding tissue was raised^37^. Currently, none of the included studies has reported the occurrence of any clinically significant adhesive complication. In an animal study conducted by Delibegovic´S and colleagues, Hem-o-lok demonstrated a lower stimulation to surrounding tissue, which resulted in less tissue adhesion, when compared with the application of Endoloop. This result provides a postulated advantage in lowering the risk of adhesive bowel obstruction^38^. Nevertheless, the long-term safety profile should still be further investigated with follow-ups on the previously included patients’ outcomes.

Apart from the safety profile, ASC with hem-o-lok also faces the pitfall of a larger working port needed. The XL size of Hem-o-lok requires a 10 mm working port, contrary to the application of an endoloop, which will only require a 5 mm working port. Thus, a larger port wound may be required for using hem-o-lok. This will require additional time for closing the fascial defect of the 10 mm port, while the 5 mm port does not necessarily require closure of the fascia^39^.

In this meta-analysis, we have demonstrated a consistently significant difference in shortening the operative time and lower chance of postoperative intra-abdominal collection by hem-o-lok with a comparable clinical profile. The price, ease of application, and promising complication profile should be considered. Further high-quality studies may provide insight into the superiority of Hem-o-Lok over endoloop ligation for appendiceal stump closure. After taking into consideration the operative cost, Hem-o-Lok is a reasonable option for ASC.

### Limitation

This meta-analysis is limited by the non-standardized surgical techniques and variation in surgeons’ experience in different studies. Different types of endoloops and operative techniques were employed. Some studies divided the appendix with vessel sealing devices while some used endo-scissors which may affect the incidence of stump leakage. These contribute to the heterogeneity between outcome measurements of the included studies. Another concern is that majority of the studies are small in sample size and the pooled result may be masked by a dominant study. This meta-analysis has tried to overcome this by performing a sensitivity test by alternatively removing the study.

Moreover, we demonstrate a reduction of operative time of 5.15 min in pooled analysis. Considered the usual operative time for laparoscopic appendicectomy being roughly around 30–40 min, this contributes up to 17% reduction in overall operative times. However, this may not lead to a clinical significance in reality given the short absolute reduction of 5 min.

## Conclusion

While both Hem-o-lok and Endoloop ligation provide a sound and promising complication profile, the application of Hem-o-Lok demonstrates a comparable if not superior to endoloop ligation in terms of operative time and potential benefit on the complication profile. When taking financial and technical aspects into consideration, it serves as a reasonable alternative to endoloop.

## Ethical approval and consent to participate

Not applicable.

## Consent

Not applicable.

## Sources of funding

The authors did not receive any funding from any party.

## Author contribution

S.H.T.P is responsible for study design, data collection, data analysis and interpretation, and writing the paper. S.Y.L. is responsible for data collection, data analysis and interpretation. A.T.Y.L. IS responsible for data analysis and interpretation.

## Conflicts of interest disclosure

The authors declare that they have no competing interests.

## Research registration unique identifying number (UIN)

Registry: Inplasy Registration number is INPLASY202360061 Hyperlink: https://inplasy.com/inplasy-2023-6-0061/

## Guarantor

Samuel Ho Ting Poon.

## Availability of data and materials

Not applicable.

## Provenance and peer review

Not applicable.

## References

[1] Lee JH Park YS Choi JS. The epidemiology of appendicitis and appendectomy in South Korea: national registry data. J Epidemiol 2010;20:97–105. 20023368 10.2188/jea.JE20090011 PMC3900807

[2] Al-Omran M Mamdani M McLeod RS. Epidemiologic features of acute appendicitis in Ontario, Canada. Can J Surg 2003;46:263–268. 12930102 PMC3211626

[3] American College of Surgeons. COVID-19 guidelines for triage of emergency general surgery patients. 2020. https://www.facs.org/for-medical-professionals/covid-19/clinical-guidance/elective-case/emergency-surgery/

[4] Poon SHT Lee JWY NG K. The current management of acute uncomplicated appendicitis: should there be a change in paradigm? A systematic review of the literatures and analysis of treatment performance. World J Emerg Surg 2017;12:46. 29075315 10.1186/s13017-017-0157-y PMC5644137

[5] Yau KK Siu WT Tang CN. Laparoscopic versus open appendectomy for complicated appendicitis. J Am Coll Surg 2007;205:60–65. 17617333 10.1016/j.jamcollsurg.2007.03.017

[6] Neugebauer EAM Troidl H Kum CK The EAES clinical practice guidelines on laparoscopic cholecystectomy, appendectomy, and hernia repair Neugebauer EAM Sauerland S Fingerhut A Millat B Buess G. EAES guidelines for endoscopic surgery. Springer. 265–289.

[7] Jaschinski T Mosch CG Eikermann M. Laparoscopic versus open surgery for suspected appendicitis. Cochrane Database Syst Rev 2018;11:CD001546. 30484855 10.1002/14651858.CD001546.pub4 PMC6517145

[8] Yaghoubian A Kaji AH Lee SL. Laparoscopic versus open appendectomy: outcomes analysis. Am Surg 2012;78:1083–1086. 23025946

[9] Jenwitheesuk K. Preliminary report: appendiceal stump management with hem-o-lock clips TM in laparoscopic appendectomy. Srinagarind Med J 2006;4:311–317.

[10] Hanssen A Plotnikov S Dubois R. Laparoscopic appendectomy using a polymeric clip to close the appendicular stump. JSLS 2007;11:59–62. 17651557 PMC3015784

[11] Di Saverio S Podda M De Simone B. Diagnosis and treatment of acute appendicitis: 2020 update of the WSES Jerusalem guidelines. World J Emerg Surg 2020;15:27. 32295644 10.1186/s13017-020-00306-3 PMC7386163

[12] Mantel N Haenszel W. Statistical aspects of the analysis of data from retrospective studies of disease. J Natl Cancer Inst 1959;22:719–748. 13655060

[13] Hasselblad VV McCrory DCD. Meta-analytic tools for medical decision making: a practical guide. Med Decis Marking 1994;15:81–96. 10.1177/0272989X9501500112 7898302

[14] Delibegović S Matović E. Hem-o-lok plastic clips in securing of the base of the appendix during laparoscopic appendectomy. Surg Endosc 2009;23:2851–2854; Epub 2009 May 14. 19440790 10.1007/s00464-009-0493-4

[15] Delibegović S. The use of a single Hem-o-lok clip in securing the base of the appendix during laparoscopic appendectomy. J Laparoendosc Adv Surg Tech A 2012;22:85–87. Epub 2011 Dec 6. 22145605 10.1089/lap.2011.0348

[16] Jenwitheesuk K Chotikawanich E Saeseow OT. Laparoscopic appendectomy: results of a new technique for stump management. J Med Assoc Thai 2012;95 (Suppl 11):S7–S10. 23961612

[17] Lucchi A Berti P Grassia M. Laparoscopic appendectomy: Hem-o-lok versus Endoloop in stump closure. Updates Surg 2017;69:61–65. 28013455 10.1007/s13304-016-0413-9

[18] Colak E Kement M Ozlem N. A comparison of nonabsorbable polymeric clips and endoloop ligatures for the closure of the appendicular stump in laparoscopic appendectomy: a prospective, randomized study. Surg Laparosc Endosc Percutan Tech 2013;23:255–258. 23751988 10.1097/SLE.0b013e31828b8382

[19] Hue CS Kim JS Kim KH. The usefulness and safety of Hem-o-lok clips for the closure of appendicular stump during laparoscopic appendectomy. J Korean Surg Soc 2013;84:27–32. Epub 2012 Dec 26. 23323232 10.4174/jkss.2013.84.1.27 PMC3539106

[20] Sadat-Safavi SA Nasiri S Shojaiefard A. Comparison the effect of stump closure by endoclips versus endoloop on the duration of surgery and complications in patients under laparoscopic appendectomy: a randomized clinical trial. J Res Med Sci 2016;21:87. 28163733 10.4103/1735-1995.192503 PMC5244687

[21] Soll C Wyss P Gelpke H. Appendiceal stump closure using polymeric clips reduces intra-abdominal abscesses. Langenbecks Arch Surg 2016;401:661–666. Epub 2016 Jun 13. 27294658 10.1007/s00423-016-1459-3

[22] Şimşek O. Comparison of endoloop and polymer locking clip in ligating appendiceal stump during laparoscopic appendectomy. Laparosc Endosc Surg Sci 2017;24:5–8.

[23] Öz B Emek E Akyüz M. Laparoscopic appendectomy using hem-O-lok polymer clips: a single-center experience. Int Surg, 2017;102:334–339.

[24] Delibegović S Mehmedovic Z. The influence of the different forms of appendix base closure on patient outcome in laparoscopic appendectomy: a randomized trial. Surg Endosc 2018;32:2295–2299. Epub 2017 Nov 2. 29098432 10.1007/s00464-017-5924-z

[25] Wilson M Maniam P Ibrahim A. Polymeric clips are a quicker and cheaper alternative to endoscopic ligatures for securing the appendiceal stump during laparoscopic appendicectomy. Ann R Coll Surg Engl 2018;100:454–458. Epub 2018 Mar 15. 29543058 10.1308/rcsann.2018.0036 PMC6111912

[26] Bhabhor VP. Appendicular stump closure by polymer clip vs endoloop in laparoscopic appendectomy. World J Lap Surg 2019;12:64–67.

[27] Ihnát P Tesař M Tulinský L. A randomized clinical trial of technical modifications of appendix stump closure during laparoscopic appendectomy for uncomplicated acute appendicitis. BMC Surg 2021;21:272. 34059039 10.1186/s12893-021-01279-z PMC8165989

[28] Mir JM Dastgeer W Quddus A. Compare the outcome of clips closure versus endoloop closure of appendicular stump. PJMHS 2021;15:745–747.

[29] Erdoğan A Türkan A. Comparison of handmade endoloop versus polymeric endoclip for stump closure in laparoscopic appendectomy. Cureus 2021;13:e16302. 34381657 10.7759/cureus.16302 PMC8352043

[30] Vinod A St B Nanda NS Ravindran GC. Comparison of two stump closure techniques in laparoscopic appendicectomy: a single-center prospective cohort study. Cureus 2022;14:e21796. 35251863 10.7759/cureus.21796 PMC8891722

[31] Mikail U Türker A Ahmet K. Comparison of endoloop and Hem-o-lok clip for stump closure in laparoscopic appendectomy: which one is more cost-effective. A retrospective study. J Exp Clin Med 2022;39:492–496.

[32] Beldi G Muggli K Helbling C. Laparoscopic appendectomy using endoloops: a prospective, randomized clinical trial. Surg Endosc 2004;18:749–750. 15026904 10.1007/s00464-003-9156-z

[33] al Fallouji M. Making loops in laparoscopic surgery: state of the art. Surg Laparosc Endosc 1993;3:477–481. 8269268

[34] Daniell JF Gurley LD Kurtz BR. The use of an automatic stapling device for laparoscopic appendectomy. Obstet Gynecol 1991;78:721–723. 1833683

[35] Nottingham JM. Mechanical small bowel obstruction from a loose linear cutter staple after laparoscopic appendectomy. Surg Laparosc Endosc Percutan Tech 2002;12:289–290. 12193829 10.1097/00129689-200208000-00019

[36] Gupta V Chauhan SPS Gupta M. Efficacy and safety of ligasure in laparoscopic sutureless appendectomy. Cureus 2022;14:e24764. 35755548 10.7759/cureus.24764 PMC9216166

[37] Bajric Amela. “Tissue reaction and the formation of adhesion after the use of DS clip in laparoscopic appendectomy. JSLS 2021;25:e2021.00063. 10.4293/JSLS.2021.00063 PMC8658051 34949906

[38] Delibegovic´ S Iljazovic´ E Katica M. Tissue reaction to absorbable endoloop, nonabsorbable titanium staples, and polymer Hem-o-lok clip after laparoscopic appendectomy. JSLS 2011;15:70–76. 21902947 10.4293/108680811X13022985131336 PMC3134701

[39] Lambertz A Stüben BO Bock B. Port-site incisional hernia—a case series of 54 patients. Ann Med Surg (Lond) 2017;14:8–11. 28119777 10.1016/j.amsu.2017.01.001 PMC5237772

